# Enhancing the Growth Rate, Biochemical Blood Indices, and Antioxidative Capacity of Broilers by Including *Aloe vera* Gel in Drinking Water

**DOI:** 10.3389/fvets.2020.632666

**Published:** 2021-01-21

**Authors:** Khairy Amber, Reayd Nofel, Reda Ghanem, Samy Sayed, Soha A. Farag, Mustafa Shukry, Mahmoud A. O. Dawood

**Affiliations:** ^1^Department of Poultry Production, Faculty of Agriculture, Kafrelsheikh University, Kafrelsheikh, Egypt; ^2^Department of Science and Technology, University College-Ranyah, Taif University, Taif, Saudi Arabia; ^3^Faculty of Agriculture, Cairo University, Giza, Egypt; ^4^Animal Production Department, Faculty of Agriculture, Tanta University, Tanta, Egypt; ^5^Department of Physiology, Faculty of Veterinary Medicine, Kafrelsheikh University, Kafrelsheikh, Egypt; ^6^Department of Animal Production, Faculty of Agriculture, Kafrelsheikh University, Kafrelsheikh, Egypt

**Keywords:** drinking water, growth rate, antioxidative response, broilers, blood health

## Abstract

Phytogenic herbal extracts received considerable attention in the broilers industry as friendly alternative substitutes to antibiotics. These additives can be included in the food or drinking water to enhance birds' growth rate and well-being. Hence, the current investigation examined the effect of including *Aloe vera* gel in drinking water on the growth rate, biochemical blood indices, and broilers' antioxidative capacity. Cobb 500 broiler chicks (*n* = 120), 1 day old of initial weight = 48.6 ± 1.65 g, were divided into three treatments where the control group was fed the basal diet without including *Aloe vera* gel in drinking water. The second and third groups were fed the basal diet, and *Aloe vera* gel was included in drinking water at 1 and 1.5%, respectively. The final body weight, weight gain, daily weight gain, and feed conversion ratio were significantly improved in birds that received drinking water with *Aloe vera* gel at 1.5% compared to the control and 1% groups (*P* ≤ 0.05). The kidney (creatinine and urea) and liver (ALT and AST) function indices of broilers that received drinking water with or without *Aloe vera* gel showed no significant differences with the control group (*P* ≥ 0.05). The blood total protein and albumin had higher values in birds that received drinking water with 1.5% *Aloe vera* gel than the control (*P* ≤ 0.05). The total blood cholesterol, triglycerides, and LDL levels were significantly decreased in the group of birds that received 1.5% *Aloe vera* gel in drinking water (*P* ≤ 0.05). The HDL level was higher in birds that received drinking water with 1.5% *Aloe vera* gel than the control (*P* ≤ 0.05). The total antioxidative capacity (TAC) and glutathione peroxidase (GPX) showed higher activity in the group of birds that received 1.5% *Aloe vera* gel while the level of malondialdehyde (MDA) was lower in birds that received drinking water with 1.5% *Aloe vera* gel than the control (*P* ≤ 0.05). In summary, including *Aloe vera* gel in drinking water enhanced the growth rate, biochemical blood indices, and broilers' antioxidative capacity.

## Introduction

Poultry production has contributed to global food security as an economical source of protein for all humanity (1). Although the production has increased recently, there is still a shortage of biosecurity, likely due to infectious diseases and feed costs (2). Additionally, birds farmed under intensive and super-intensive systems are susceptible to contagious diseases due to the spread of pathogens and invaders' emergence, which induces severe mortality and economic loss (3). Subsequently, common integrated approaches by applying antibiotic drugs, vaccines, and therapeutics must be used (4). However, chemotherapies are no longer allowed in many countries due to their environmental and hazardous impacts (e.g., the spread of antimicrobial-resistant bacteria, the weakening of birds' immunity, and human health safety concerns) (5). Accordingly, safer alternative substances are highly recommended to control the infection pathogens.

Medicinal plant metabolites and their bioactive compounds have a broad therapeutic range to promote health, intestinal microflora, antioxidant effects, and immune system parameters in poultry (6). Phytogenics and herbals and their extracts and essential oils manifested appetite-stimulating, growth-promoting, and immunostimulatory activities in poultry production (7). A particular focus has been given to *Aloe vera* as natural immunostimulants and antioxidant agents in poultry production (8). *Aloe vera* is a tropical plant belonging to the Liliaceae family and is well-known for its therapeutic and remedial properties associated with its content of bioactive components (9). It has abundant amounts of polysaccharides (e.g., acemannan), which act as immunomodulatory and antibacterial agents against harmful bacteria. *Aloe vera* gel is resistant to high acidity in birds' intestines, which guarantees its efficacy and influence (10). *Aloe vera* gel has an antibacterial effect to kill pathogenic microorganisms by breaking down their cell walls and weakens their activity and allowing the beneficial microorganisms to show its effect on the digestion of nutrients (11). Also, *Aloe vera* gel enhances the permeability of absorbed nutrients through the intestinal barriers. Accordingly, the potential impact of *Aloe vera* gel on the immune system is attributed to the enhanced local intestinal immunity (12). Further, *Aloe vera* gel has abundant amounts of polyphenols and natural antioxidants that scavenge the overproduction of free radicals that induce lipid peroxidation and immune cells' damage (13). Additionally, the application of *Aloe vera* gel as feed additives enhanced the immunity of broilers and was suggested as a replacer for antibiotics (14, 15).

The most appropriate feed additive strategy is the oral administration through bird feed, which markedly improves the performances (16). The ultimate beneficial effects of *Aloe vera* gel included in drinking water are not well-described (17). In this sense, Shokraneh and Ghalamkari (18) reported that *Aloe vera* gel could be successfully added to broilers' drinking water with the possibility of enhancing growth performance, immunity, and resistance to pathogenic bacteria. Hence, this study aims to illustrate the effects of *Aloe vera* gel included in the drinking water of poultry on the growth performances, biochemical blood indices, and antioxidant capacity.

## Materials and Methods

This study was carried out at the poultry research farm, Department of Poultry Production, Faculty of Agriculture, Kafrelsheikh University. The chemical and biochemical analyses were done in Laboratories of the Animal Production Research Institute, Ministry of Agriculture, Egypt. The experiments were carried out following the Ethics Committee of local experiment animal care guidelines at the Faculty of Agriculture, Kafrelsheikh University, Egypt.

### Experimental Diets

Diets were formulated to meet or exceed broilers' requirements as declared by the National Research Council (19) (Table 1). A phase feeding program was adopted: starter (1–10 days), grower (11–24 days), and finisher (25–32 days) (Table 1) were provided to the broilers.

**Table 1:** The formulation and chemical composition of the basal diet.

**Ingredients (g/kg)**	**Starter**	**Grower**	**Finisher**
Yellow corn	575	615	650
Soybean meal, 44%	320	278	240
Corn gluten meal, 60%	58	50	40
Limestone	13.9	13.2	12.5
Di-calcium phosphate	15	15	15
NaCl	3	3	3
DL-methionine	1.85	1.8	1.65
L-lysine HCl	2.75	3.5	4.35
Plant oil	7.5	17.5	30.5
Premix^*^	3	3	3
Total (kg)	1,000	1,000	1,000
Nutrient levels %^**^			
ME (MJ/kg)	12.43	12.85	13.15
Crude protein	23	21	19
Calcium	1.0	0.94	0.91
Available phosphorus	0.46	0.43	0.43
Digestible Thr	0.77	0.70	0.63
Digestible Met + Cysteine	0.84	0.78	0.71
Digestible Lys	1.19	1.14	1.11

* *Vitamin and mineral premix were added as 3 kg/ton of diet: Vitamin A (6,000,000 IU); Vitamin D_3_. (900,000 IU); Vitter (40,000 mg); Vitamin k (2,000 mg); Vitamin B_1_ (2,000 mg); Vitamin B_2_ (4,000 mg); Vitamin B_6_ (2,000 mg); Vitamin B_12_ (10 mg); Niacin (50,000 mg); Folic acid (3,000 mg); Pantothenic acid (10,000 mg); Biotin (50 mg); Choline (250,000 mg); Copper (50,000 mg); Iron (50,000 mg); Manganese (8,500 mg); Zinc (50,000 mg); Iodine (200 mg); Selenium (100 mg); and Cobalt (100 mg)*.

** *Calculated according to NRC (19) and formulated to contain the National Research Council (19) requirements of all nutrients*.

### *Aloe vera* Source

*Aloe vera* gel extracted from fresh Aloe leaves (*Aloe barbadensis* Miller) was prepared by following Shokraneh and Ghalamkari (18). Fresh leaves were gathered from the local garden for the extraction of gel. The leaves were cleaned with water, and the Aloe gel was extracted from the leaf manually by making a cut. Latex of the leaf was removed, and the gel was collected in a beaker. A 10% (w/v) concentrated infusion was prepared by taking 100 g of fresh gel in a glass bottle, and a liter of boiled distilled water was poured on it. The bottle was shaken for 5–7 min to ensure thorough mixing and kept for 6–8 h at room temperature before use.

### Experimental Birds

Cobb 500 broiler chicks (*n* = 120), 1 day old of initial weight = 48.6 ± 1.65 g, were divided into three treatments; each included four replicates of 10 chicks (floor pens; 10 birds/m^2^) placed inside a room equipped with a chain feeder system and automatic nipple cup drinker. Chicks were raised in a windowed experimental farm. The temperature inside the barn was maintained at around 32 to 34°C from day 1 to day 5 post-hatch and gradually decreased to 24°C at 21 days with relative humidity from 50 to 60% throughout the experiment (35 days). All birds were kept under the same managerial conditions. Feed and water were offered *ad libitum*. Light was provided the whole day with only 1 h cutoff to get them used to the darkness. The photoperiod was maintained as a 21-h light/3-h dark cycle. The control group was fed the basal diet without including *Aloe vera* gel in drinking water. The second and third groups were fed the basal diet, and *Aloe vera* gel was included in drinking water at 1 and 1.5%, respectively.

### Productive Performance of Broiler Chicks

The individual body weight and feed consumption were recorded every week during the experimental period. At the final stage of research (35 days), growth performance and feed conversion ratio (FCR) were determined by the following formula:

FCR = Feed intake (g)/Live body weight (g)

Live body weight gain (g) was calculated by subtracting the live weight at the beginning of the experiment from the live body weight at the final stage of the experiment. Mortality and the clinical health status of all birds were monitored daily, and the mortality percentage was calculated as follows:

Mortality % = Number of dead animals/Total number of animals at start × 100.

### Blood Sampling and Chemical Analysis

At 35 days, blood samples from 18 birds (1 bird per replicate; 6 birds per treatment) were collected from the wing vein immediately before slaughter. Blood samples were collected from the wing and jugular veins using insulin syringes (28-gauge needle) under diethyl ether sedation. Blood samples were collected in tubes without anticoagulant for serum collection. Blood samples were centrifuged at 1,500 × *g* for 15 min for serum separation and kept at −20°C to be used for determination of the following serum parameters: cholesterol, triglyceride (TG), low- and high-density lipoprotein (LDL and HDL), total protein, albumin, aspartate aminotransferase (AST), alanine aminotransferase (ALT), urea, and creatinine. These parameters were measured by a spectrophotometer using standard commercial kits (Biodiagnostic Co., Egypt) according to the manufacturer's instructions. The globulin content was calculated mathematically from the difference between total protein and albumin.

### Serum Total Antioxidant Capacity

The glutathione peroxidase (GPX) and total antioxidant capacity (20) were analyzed by using Biomerieux Kits (Biomerieux, France) according to the manufacturer's instructions. The concentration of malondialdehyde (MDA) in the serum was determined, according to Nair and Turner (21).

### Statistical Analysis

Data for all the response variables were subjected to a one-way analysis of variance (SAS, 2,000). Variables having a significant *F*-test (*P* ≤ 0.05) were compared using Duncan's Multiple Range-Test. Model: *X*_*ij*_ = μ + *Ti* + *e*_*ij*_

Where *X*_*ij*_ = any observation, μ = overall mean, *Ti* = Treatments (*i* = 1, 2,…and 10), *e*_*ij*_ = Experimental error.

## Results

### Growth Performance

The final body weight, weight gain, and daily weight gain significantly increased in birds that received drinking water with *Aloe vera* gel at 1.5% compared to the control and 1% groups (*P* ≤ 0.05; Table 2). The FCR recorded the lower value in the group of birds that received water with 1.5% *Aloe vera* gel than the control without significant difference with those that received 1% *Aloe vera* gel (*P* ≤ 0.05; Table 2). The daily feed intake was not altered by including *Aloe vera* in drinking water. Interestingly, the mortality rate of the birds that received 1 or 1.5% *Aloe vera* gel was lower than the control group (*P* ≤ 0.05; Table 2).

**Table 2:** Effect of *Aloe vera* gel on growth performance of broiler chicken.

**Parameters**	***Aloe vera*** **gel level (%)**			**SEM**	***P*-value**
	**0**	**1.0**	**1.5**		
Initial body weight (g)	48.61	48.33	48.86	0.445	0.9993
Final body weight at 35 days (g)	1824.9^b^	1888.1^b^	1981.2^a^	32.32	0.0067
Weight gain (g) (1–35 days)	1776.1^b^	1839.3^b^	1932.4^a^	0.921	0.0067
Daily weight gain (g) (1–35 days)	50.8^b^	52.6^b^	55.2^a^	0.921	0.0067
Feed intake (g/day) (1–35 days)	79.7	77.0	77.9	1.139	0.4677
Feed conversion ratio (1–35 days)	1.57^a^	1.46^ab^	1.41^b^	0.039	0.0694
Mortality (%)	5.0^a^	2.5^b^	2.5^b^	-	-

*Means in the same row with different superscripts are significantly different (P ≤ 0.05; N = 40). SEM, Standard error of means*.

ab *refer to that no significances with the groups has a or b superscript*.

### Blood Biochemical Variables

**Figure 1:**
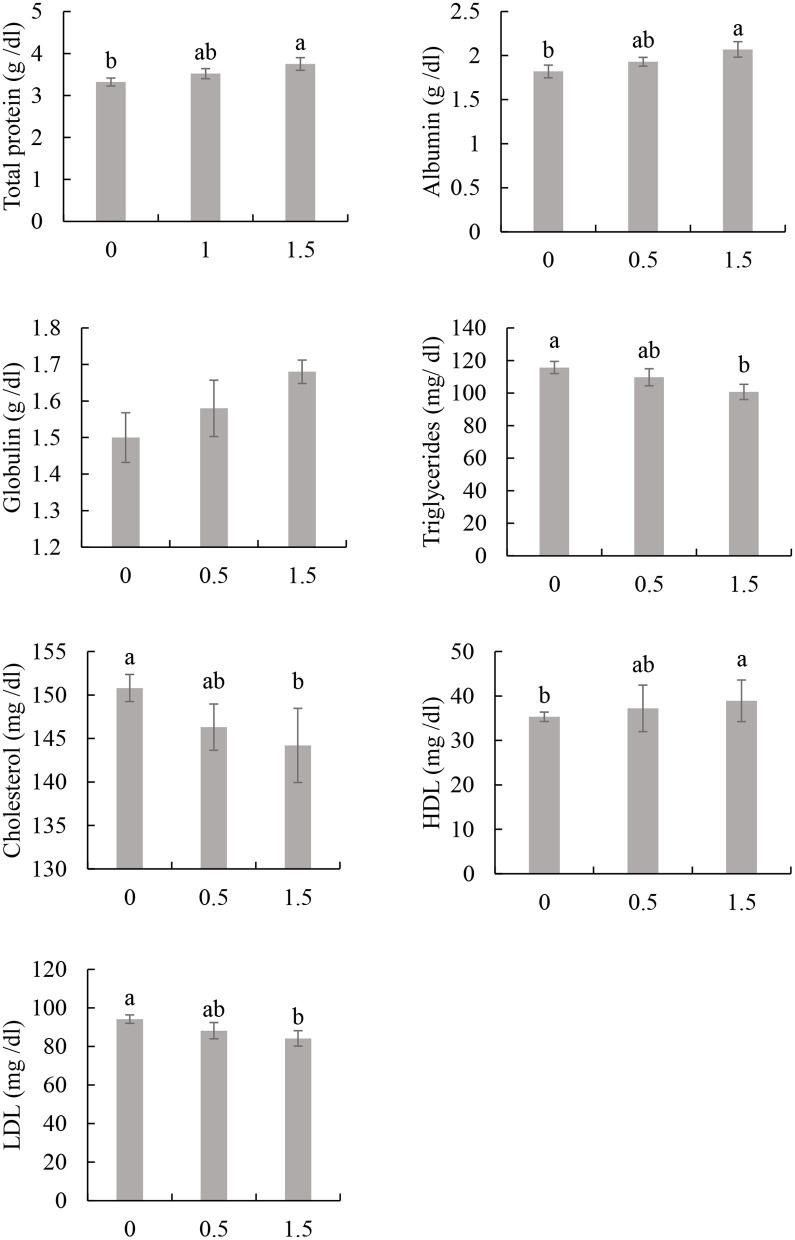
Effect of *Aloe vera* gel included in drinking water on the biochemical blood indices of broiler chicken: cholesterol, triglyceride, low- and high-density lipoprotein (LDL and HDL), total protein, albumin, and globulin. Bars with different superscripts are significantly different (*P* ≤ 0.05; *N* = 4).

The kidney (creatinine and urea) and liver (ALT and AST) function indices of broilers that received drinking water with or without *Aloe vera* gel showed no significant differences with the control group (*P* ≥ 0.05; Table 3). The blood total protein and albumin had higher values in birds that received drinking water with 1.5% *Aloe vera* gel than the control (*P* ≤ 0.05) without significant differences with those that received 1% *Aloe vera* gel in drinking water (*P* ≥ 0.05; Figure 1). However, the blood globulin was not impacted by the inclusion of *Aloe vera* gel in drinking water.

**Table 3:** Effect of *Aloe vera* gel on the kidney and liver function of broiler chicken.

**Parameters**	***Aloe vera*** **gel level (%)**			**SEM**	***P*-value**
	**0**	**1.0**	**1.5**		
**Kidney function**					
Creatinine (mg/dl)	0.57	0.58	0.58	0.017	0.8880
Urea (mg/dl)	11.47	11.42	11.35	0.357	0.9366
**Liver function**					
AST (U/L)	257.2	264.3	265.3	13.71	0.9704
ALT (U/L)	56.8	56.3	55.2	2.620	0.8920

*Means in the same row with different superscripts are significantly different (P ≤ 0.05; N = 4). SEM, Standard error of means; AST, Aspartate aminotransferase; ALT, alanine aminotransferase*.

The total blood cholesterol, triglycerides, and LDL levels significantly decreased in the group of birds that received 1.5% *Aloe vera* gel in drinking water compared to those of the control group (*P* ≤ 0.05; Figure 1). No significant differences were observed between the groups of 1 and 1.5% in terms of total cholesterol, triglycerides, and LDL levels (*P* ≥ 0.05). The level of HDL was higher in birds that received drinking water with 1.5% *Aloe vera* gel than the control (*P* ≤ 0.05) without significant differences with those that received 1% *Aloe vera* gel in drinking water (*P* ≥ 0.05; Figure 1).

### Antioxidative Response

**Figure 2:**
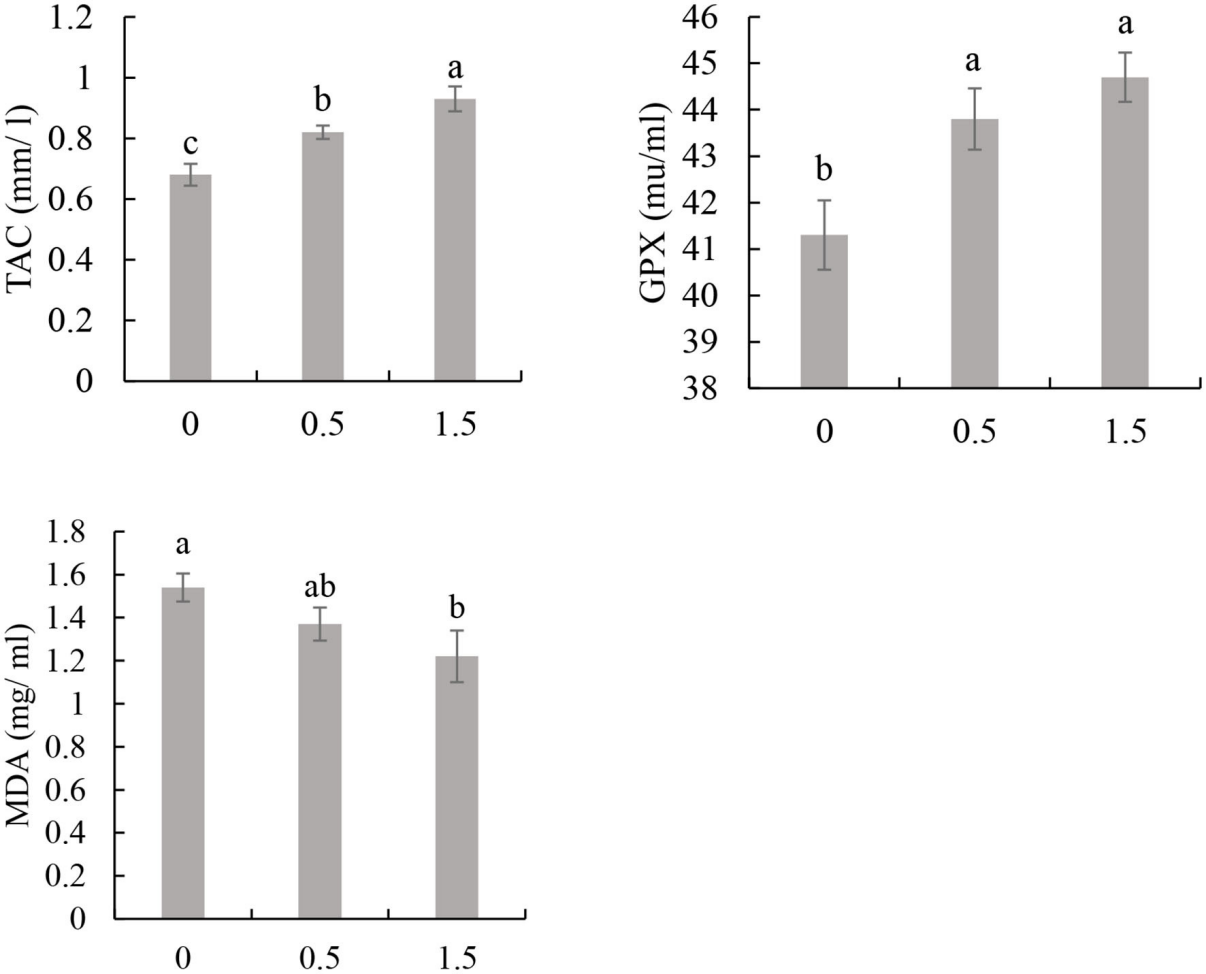
Effect of *Aloe vera* gel included in drinking water on the blood antioxidative responses of broiler chicken: glutathione peroxidase (GPX), total antioxidant capacity (TAC), and malondialdehyde (MDA) level. Bars with different superscripts are significantly different (*P* ≤ 0.05; *N* = 4).

The total antioxidative capacity (TAC) showed higher activity in birds that received 1.5% *Aloe vera* gel than the other groups. Simultaneously, the group of birds that received 1% *Aloe vera* gel had higher TAC than the control (*P* ≤ 0.05; Figure 2).

The activity of GPX was significantly enhanced in the group of birds that received 1 or 1.5% of *Aloe vera* gel in drinking water compared to the control group (*P* ≤ 0.05; Figure 2).

The level of MDA was lower in birds that received drinking water with 1.5% *Aloe vera* gel than the control (*P* ≤ 0.05), without significant differences with those that received 1% *Aloe vera* gel in drinking water (*P* ≥ 0.05; Figure 2).

## Discussion

Therapeutics are no longer recommended for poultry production, and their usage is restricted in several countries, especially for organic food production (22). Phytogenic additives are applied in the modern poultry industry for their beneficial and promoting influences on the growth performance, immune response, and resistance against farming stressors (7). Several studies were conducted to investigate the potential role of *Aloe vera* in poultry production as feed additives or through drinking water (8). The obtained results showed marked growth performance, feed efficiency, biochemical blood indices, and oxidation resistance. Concurrent with the previous studies, the present study showed enhanced growth rate, feed utilization, and immune response in birds treated with *Aloe vera* gel in drinking water. The present study showed enhanced antioxidative condition and blood biochemical derivatives, which was not evaluated in the previous studies.

The results showed an enhanced growth rate and FCR in broilers treated with *Aloe vera* (1.5%) in drinking water at the end of the trial (35 days). In a similar sense, Shokraneh and Ghalamkari (18), Olupona and Omotoso (23), and Wang and Jia (24) reported that poultry treated with *Aloe vera* gel in drinking water had enhanced growth performance. Likewise, Odo and Ekenyem (25), Mmereole (26), and Wang and Jia (24) illustrated that poultry fed dietary *Aloe vera* as feed additives had enhanced growth rate and feed utilization. The enhanced growth performance can be attributed to the role of *Aloe vera* in increasing the abundance of beneficial bacteria and reducing the harmful bacteria. In this sense, Shokraneh and Ghalamkari (18) concluded that broilers treated with *Aloe vera* in drinking water had an increased number of *Lactobacillus* bacteria, which are known for their role in enhancing the digestion and absorption of nutrients in the gut of birds. Correspondingly, the number of pathogenic bacteria decreases and the birds can efficiently utilize the feed and nutrients. The enhanced growth rate can also be elucidated to the composition of *Aloe vera*, which has abundant amounts of polysaccharides, vitamins, minerals, and organic acids (3), which can enhance the feed utilization and, in turn, the growth rate.

Blood biochemical indices are bio-indicators associated with the general health condition, metabolism, and birds' well-being (1). They indicate the immunity status and metabolic rate (total protein and albumin), kidney function (urea and creatinine), liver health (ALT and AST), and the level of fat metabolism in the blood. The levels of these derivatives should be within the normal range for healthy birds, and over or lower levels refers to the unstable health condition. The present study results presented enhanced blood total protein and albumin levels in birds treated with *Aloe vera* gel in drinking water. The blood total protein and albumin had higher values in birds treated with 1.5% *Aloe vera* gel than the control, which contradict Singh and Koley (27) and Sharma and Singh (13), who stated no significant differences among the birds fed dietary *Aloe vera*. The contradicting results are probably attributed to the differences in the addition of *Aloe vera* wherein it was supplied in drinking water in the present study while Singh and Koley (27) and Sharma and Singh (13) included *Aloe vera* as dietary additives. Another reason may be associated with the difference in the applied levels of *Aloe vera* and the strain of birds. Generally, the present study supported the theory that considers oral administration of phytogenic additives through rearing water is more effective than including them in birds' feed.

The levels of triglycerides, total cholesterol, and LDL were decreased while the HDL level was increased in birds treated with *Aloe vera* gel in drinking water. In line with the present study, Naghi Shokri and Ghasemi (11) illuminated that poultry fed dietary *Aloe vera* had reduced total blood cholesterol and LDL levels. However, no previous studies were done to reveal the impact of *Aloe vera* included in drinking water on broilers' blood lipids. *Aloe vera* gel has acemannan, which is the main polysaccharide that can modify blood cholesterol by regulating fat metabolism in the liver (28). Misawa and Tanaka (29) illustrated that *Aloe vera* could reduce the level of lipids in the blood by enhancing the sensitivity of cells to insulin, which led to the reduction of free fatty acids released from fat tissue to the blood.

The kidney function is evaluated by the measurement of creatinine and urea levels in the blood, and the results showed no marked changes among the birds treated with varying levels of *Aloe vera* gel. The results also showed that the liver function, as shown by the activities of ALT and AST enzymes, did not deteriorate by including *Aloe vera* gel in drinking water. Concurrent with the present study, Sharma and Singh (13) stated that broilers treated with *Aloe vera* had no ALT changes as an indicator of liver function. Based on the obtained results, birds treated with *Aloe vera* gel in drinking water did not suffer from stressors that can impair the kidney and liver functions.

Broilers usually suffer from oxidative stress induced by heat stress, intensive farming, and infectious diseases (30, 31). The oxidative stress resulted from the overproduction of free radicals and the disfunction of antioxidative defenses to degenerate these radicals (32). It causes lipid peroxidation and DNA damage in the cells of birds' body, including the immune defense cells, and can be measured by the MDA level (33). Several antioxidant responses defend the cell from oxidation, including the GPX generation and the TAC (2). The results showed that birds treated with *Aloe vera* gel in drinking water had enhanced GPX and TAC with reduced MDA, which refers to enhanced antioxidative capacity. The activated antioxidative status in broilers is attributed to the content of *Aloe vera* from the natural antioxidants that can degenerate the free radicals leading to a natural antioxidative response (34). Although no studies were made on the role of *Aloe vera* in enhancing the antioxidative response of broilers, the obtained results are in line with several results that proved the potential role of phytogenic additives in improving the antioxidative capacity of birds (35, 36).

## Conclusion

The current study results illustrated that the inclusion of *Aloe vera* gel in broilers' drinking water is recommended to enhance the growth performance and feed utilization. Additionally, birds showed enhanced blood biochemical profiles of healthy liver and kidney. The blood total protein and albumin values had improved on birds that received drinking water with 1.5% *Aloe vera* gel. The total blood cholesterol, triglycerides, and LDL levels were decreased in the group of birds that received 1.5% *Aloe vera* gel in drinking water. The TAC and the GPX showed improved activity while the level of MDA was decreased in birds that received 1 or 1.5% *Aloe vera* gel. Interestingly, the present study reported the role of *Aloe vera* in enhancing the antioxidative capacity of broilers.

## Data Availability Statement

The raw data supporting the conclusions of this article will be made available by the authors, without undue reservation.

## Ethics Statement

The animal study was reviewed and approved by Faculty of Agriculture, Kafrelsheikh University, Kafrelsheikh, Egypt.

## Author Contributions

KA, RN, RG, and MD: conceptualization. KA, RN, and RG: formal analysis. KA, RG, SS, and MD: funding acquisition. KA, RN, RG, MS, and MD: methodology. KA, RN, RG, and MD: project administration. KA and RN: supervision. KA, SS, SF, MS, and MD: writing—original draft. All authors contributed to the article and approved the submitted version.

## Conflict of Interest

The authors declare that the research was conducted in the absence of any commercial or financial relationships that could be construed as a potential conflict of interest. The handling editor declared a past co-authorship with one of the authors MD.
